# Laser-printed document classification using random forest and gray prediction models

**DOI:** 10.1016/j.isci.2025.114131

**Published:** 2025-11-21

**Authors:** Yinxuan Qu, Chenyang Yu, Chunhui Li, Yuzhu Yang

**Affiliations:** 1 School of Investigation, People’s Public Security University of China, Beijing, China; 2 School of Information Network Security, People’s Public Security University of China, Beijing, China; 3 Key Laboratory of the Questioned Document Examination (China Criminal Police University), Ministry of Public Security of China, Shenyang, China

**Keywords:** applied sciences, network

## Abstract

This paper presents a classification method for laser-printed documents, integrating the random forest algorithm with the gray prediction model to enhance the accuracy and reliability of forensic document examination. The study utilizes 14 laser printers from five different brands as experimental subjects and extracts 14 key feature parameters such as gray mean, contrast, and distribution symmetry using the ImageXpert analysis system. Classification is done by the random forest algorithm, and the gray prediction model is used to enhance accuracy of classification. Finally, experimental results show that the proposed method achieves high precision or accuracy (96.00% for Chinese characters with fewer strokes and 92.86% for punctuation marks [periods]) for the character and punctuation classification. Compared to traditional classification methods, this approach exhibits superior stability and accuracy. The findings highlight the advantages of non-destructive analysis, efficient classification, and robustness, underscoring its potential as a valuable technological tool for forensic document examination in legal contexts.

## Highlights

• Integrates random forest and gray prediction for printer source classification
• Hybrid model achieves 96.00% accuracy for Chinese characters and 92.86% for punctuation
• Offers a non-destructive, efficient, and stable document analysis methodology
• Enhances forensic traceability of printed documents with a data-driven methodology

## Graphical abstract

**Figure:**
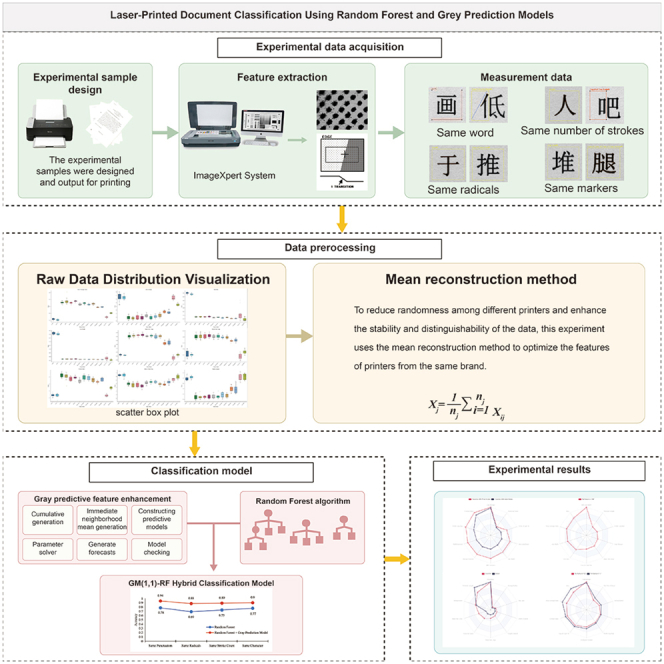

## Introduction

With the rapid advancement of information technology, digital office environments have become the dominant mode of modern workplaces. In this context, laser printers have emerged as the preferred choice in the office equipment market due to their high printing speed, stable imaging quality, and cost-effectiveness. Over the past few years, along with the far-spread use of digital technologies, were also growing the use of printed documents in the criminology and in the economy. Forensic document examination is often made difficult by the fact that perpetrators often try to avoid legal consequences by skillfully creating or slightly altering printed documents.

Although laser printers are manufactured to standardized specifications, subtle variations between individual units still occur during their operational use. These differences, which can arise from factors such as mechanical alignment, fuser temperature, and the condition of the photoconductive drum, gradually accumulate over time. As a result, printers may exhibit unique, identifiable characteristics in the printed output. Such distinctive features are essential in forensic document analysis, as they enable examiners to link printed materials to a specific printer, providing valuable evidence in legal investigations. While modern printers are generally reliable, defects and wear that develop over time can affect print quality and present challenges for forensic analysis. Traditional methods of identifying printed documents—such as chemical toner analysis and morphological examination of toner traces—are heavily reliant on the expertise of the examiner and are prone to subjectivity.^1^^,^^2^^,^^3^ Therefore, there is an urgent need for more objective, scientifically validated approaches to enhance the reliability and accuracy of document examination.

In this context, printing quality evaluation methods based on mathematical image analysis have gradually emerged as promising alternatives.For instance, features like line width, roughness, dot roundness and ink blemishes were measured by John Oliver et al. using the ImageXpert software. For inkjet prints, they showed that line roughness, dot roundness, and number and area of ink splashes around characters distinguished their study.^4^ Liang Zheng et al. used ImageXpert to extract grayscale eigenvalues from printed and copied documents produced by a Ricoh MP4000BP laser printer-copier, finding stable and statistically significant differences between the two types.^5^ Xiaobin et al. also conducted a large-scale study with 18 laser printers from nine major brands, extracting 116 parameters and achieving 99.8% classification accuracy using the C4.5 decision tree algorithm.^6^ Han Xingzhou et al. further explored the stability of grayscale features in continuously printed documents, identifying highly stable indicators that could differentiate outputs from the same printer.^7^

Beyond ImageXpert-based feature measurement, recent years have witnessed rapid advances in algorithmic approaches for source printer identification, particularly in the integration of deep learning into forensic document analysis. Bibi et al. proposed a text-independent method leveraging transfer learning on pre-trained convolutional neural networks (CNNs) to extract patch-level texture features, achieving 95.52% accuracy, which further increased to 98.06% when combined with character-level features.^8^ Guo et al. introduced an enhanced CNN architecture (SE-BRB-Net) incorporating bottleneck residual blocks and squeeze-excitation attention modules, reaching 98.77% accuracy across nine printers.^9^ Ferreira et al. applied Siamese neural networks to frame printer identification as a verification problem, reporting 97% accuracy in closed-set and 86% in open-set scenarios.^10^^,^^11^^,^^12^ Joshi et al. developed the printer-specific local texture descriptor, maintaining high accuracy across font variations,^13^^,^^14^ while Navarro et al. integrated convolutional texture gradient filters with random forest (RF) classifiers to achieve both high classification performance (96.53%) and interpretable feature visualization, a quality valued in forensic science.^15^

Despite these advances, most deep learning-based methods rely on large annotated datasets and substantial computational resources, which are not always available in forensic practice. Traditional machine learning approaches, while more resource-efficient and interpretable, can suffer from reduced stability and generalization in small sample, noisy conditions typical of real-world casework. To address this gap, the present study proposes a hybrid classification framework combining the gray prediction model (GM[1,1]) with the RF algorithm. By leveraging gray prediction to enhance the stability of multidimensional features—such as mean grayscale value, contrast, and distribution symmetry—extracted via ImageXpert, and then classifying with a robust ensemble method, the GM(1,1)-RF approach achieves high accuracy even with limited training data. This method offers an objective, precise, and computationally efficient technical pathway for laser-printed document source identification, meeting the evidentiary requirements of modern forensic science.

## Results

### Raw data distribution visualization

**Figure 1:**
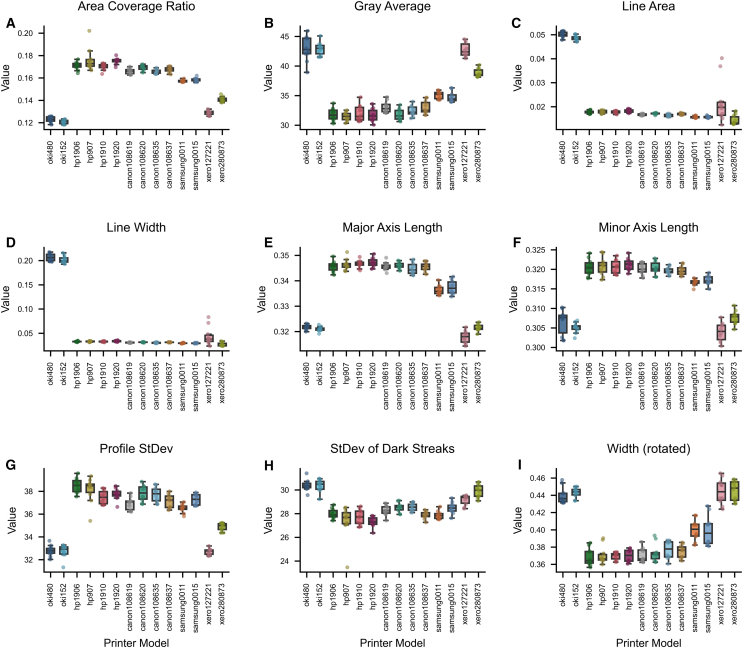
An example of a scatter boxplot of the same character (A–I) Scatter boxplots of nine key feature parameters for the same Chinese character printed by different laser printer models: (A) area coverage ratio, (B) gray average, (C) line area, (D) line width, (E) major axis length, (F) minor axis length, (G) profile standard deviation, (H) standard deviation of dark streaks, and (I) width (rotated).

To gain a deeper understanding of the distribution and differences in key feature parameters across printers, we first conducted statistical descriptions and visualizations of the raw data. Using the same Chinese character “画”as an example, Figure 1 presents scatter boxplots of nine key feature parameters (such as area coverage ratio, gray average, etc.) measured on the same character by different laser printer brands. These plots provide an intuitive view of the value distribution, quartiles, and outliers for each printer on these parameters. Preliminary statistical analysis and visualizations indicate that significant differences exist among printers in several key features, which provides a solid foundation for the construction of subsequent classification models.

### Analysis of experimental results for the same markers

In the punctuation classification experiments, it is found that the model can efficiently discriminate the different brands of the punctuation marks with high classification accuracy. In particular, the difference of feature parameter (e.g., roundness, perimeter length and vertical extent) between categories is notable.

**Figure 2:**
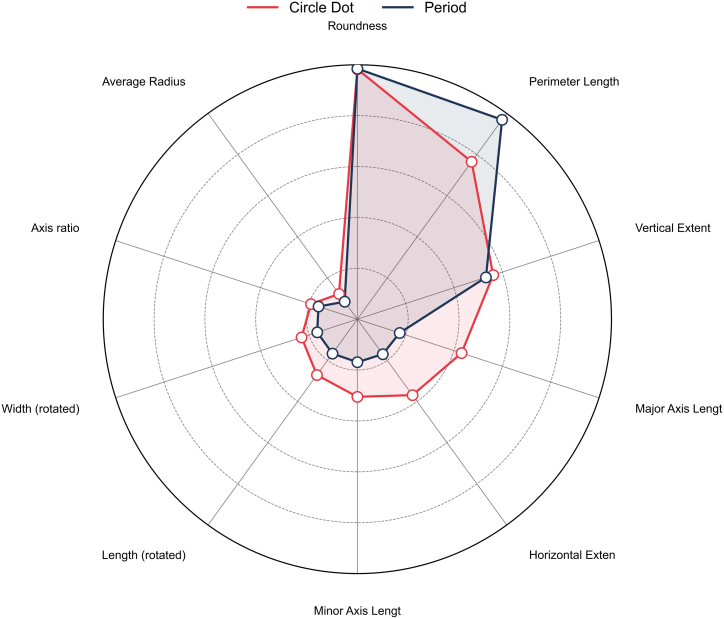
Feature importance analysis of punctuation marks

The model’s predictions are entirely correct for most of these categories, since most of the brands in the test set have a precision and recall of 1.00. However, a slight decrease in F1-score is observed for individual categories, such as Canon (F1-score = 0.82) and HP (F1-score = 0.80). The detailed classification results are presented in Table 1, and the feature importance analysis is shown in Figure 2.

**Table 1:** Classification results of punctuation marks

Training Set				Test Set		
Category	Precision	Recall	Recall	Precision	Recall	Recall
Oki	1.00	1.00	1.00	1.00	1.00	1.00
Samsung	1.00	1.00	1.00	1.00	1.00	1.00
Canon	1.00	1.00	1.00	0.78	0.88	0.82
Epson	1.00	1.00	1.00	0.93	0.75	0.80
Xerox	1.00	1.00	1.00	1.00	1.00	1.00
Average	1.00	1.00	1.00	0.94	0.93	0.92

### Analysis of experimental results for the same partials

In the classification experiment of the same radicals, the common radicals “口” and “亻” were selected for analysis. The experimental results indicate that gray average and area are the two key parameters. These parameters exhibit a high degree of differentiation among printouts from different printer brands.

**Figure 3:**
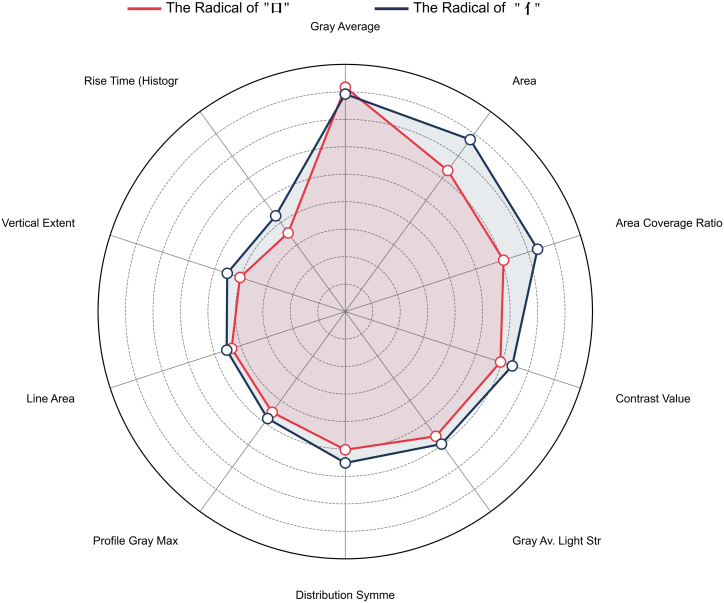
Importance of feature differentiation for the same radicals

Although the overall classification performance is high, the recall for certain brands has decreased. For instance, in the classification task for the character “口”, the Samsung brand has a recall of only 0.33, whereas the HP brand achieves a recall of 1.00. This suggests a degree of imbalance in the printing characteristics among different brands. The detailed classification results are presented in Table 2, and the feature differentiation importance is shown in Figure 3.

**Table 2:** Classification results for the same radicals

“口”Training Set				“口”Test Set		
Category	Precision	Recall	F1-Score	Precision	Recall	F1-Score
Oki	1.00	1.00	1.00	0.89	1.00	0.94
Samsung	1.00	1.00	1.00	0.75	0.33	0.46
Canon	1.00	1.00	1.00	0.80	0.80	0.80
Epson	1.00	1.00	1.00	0.67	1.00	0.80
Xerox	1.00	1.00	1.00	1.00	0.80	0.89
Average	1.00	1.00	1.00	0.82	0.79	0.78

**“亻”Training Set**				**“亻”Test Set**		

**Category**	**Precision**	**Recall**	**F1-Score**	**Precision**	**Recall**	**F1-Score**

Oki	1.00	1.00	1.00	1.00	0.38	0.55
Samsung	1.00	1.00	1.00	1.00	0.56	0.71
Canon	1.00	0.83	0.91	0.67	0.40	0.50
Epson	0.86	1.00	0.92	0.56	1.00	0.71
Xerox	1.00	1.00	1.00	0.50	1.00	0.67
Average	0.97	0.97	0.97	0.74	0.67	0.63

### Analysis of experimental results for the same number of strokes

In the classification experiments with the same number of strokes, the model demonstrates good classification ability by utilizing features such as gray average, distribution symmetry, and contrast value. For instance, in the task of classifying 12-stroke Chinese characters, most brands achieve an F1-score close to 1.00. However, the Canon (F1-score = 0.55) and HP (F1-score = 0.44) brands show relatively lower recall.

**Figure 4:**
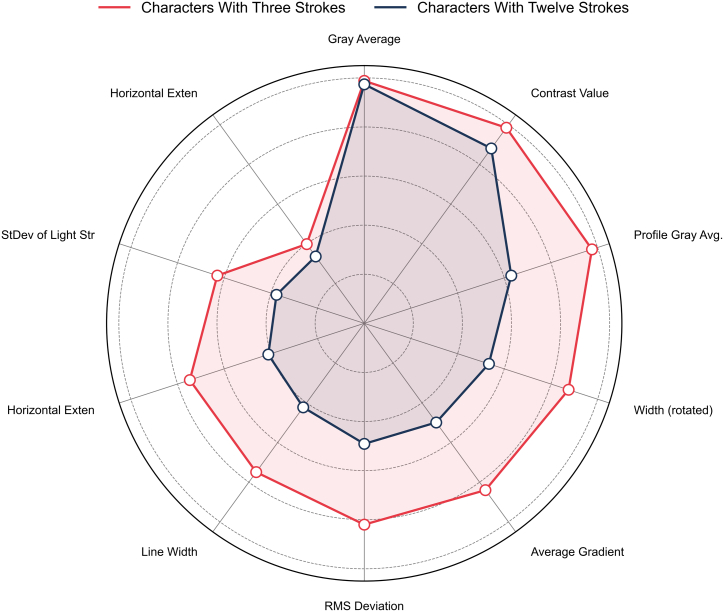
Importance of feature differentiation for Chinese characters with the same number of strokes

In contrast, for the classification task involving 3-stroke Chinese characters, the model exhibits near-perfect performance on the training set. Nevertheless, on the test set, the recall for Samsung drops to 0.50, while the F1-score for Canon decreases to 0.80. This indicates that for Chinese characters with fewer strokes, there is considerable overlap in print features among certain brands, which hampers the model’s discriminative capability. As evidenced in Figure 4, key features like gray average and distribution symmetry show lower differentiation power for complex glyphs.

The accuracy rates for each classification task are detailed in Table 3, while the importance of feature parameters is illustrated in Figure 4.

**Table 3:** Classification results for Chinese characters with the same number of strokes

Three-Stroke Character Training Set				Three-Stroke Character Test Set		
Category	Precision	Recall	F1-Score	Precision	Recall	F1-Score
Oki	1.00	1.00	1.00	1.00	1.00	1.00
Samsung	1.00	1.00	1.00	1.00	0.50	0.67
Canon	1.00	1.00	1.00	0.67	1.00	0.80
Epson	1.00	1.00	1.00	1.00	1.00	1.00
Xerox	1.00	1.00	1.00	1.00	1.00	1.00
Average	1.00	1.00	1.00	0.93	0.90	0.89

**Twelve-Stroke Character Training Set**				**Twelve-Stroke Character Test Set**		

**Category**	**Precision**	**Recall**	**F1-Score**	**Precision**	**Recall**	**F1-Score**

Oki	1.00	1.00	1.00	1.00	0.75	0.86
Samsung	1.00	1.00	1.00	1.00	1.00	1.00
Canon	1.00	1.00	1.00	0.50	0.60	0.55
Epson	1.00	1.00	1.00	0.50	0.40	0.44
Xerox	1.00	1.00	1.00	0.67	1.00	0.80
Average	1.00	1.00	1.00	0.73	0.75	0.73

### Analysis of results for the same word

In the classification experiments for the same characters, root mean square (RMS) line width, area, and area coverage ratio emerged as the three most discriminative feature parameters. These parameters exhibit consistent variability across samples from different printer brands, making them crucial for classification.

The experimental results indicate that, while the F1-score for most categories remains close to 1.00, there are slight declines for specific brands. Notably, the F1-score for Canon decreases to 0.82, while the F1-score for HP drops to 0.80. This suggests that the model still encounters misclassification issues for certain printing samples from these brands.

**Figure 5:**
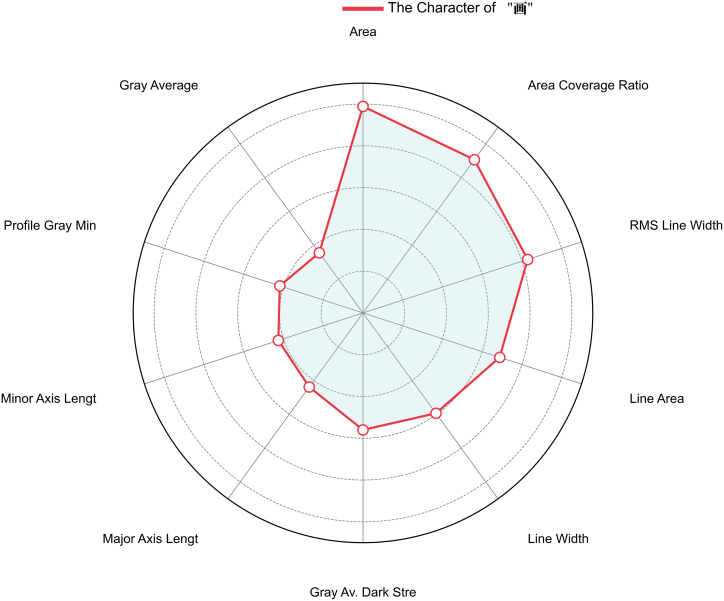
Importance of feature differentiation for the same word

The detailed classification results are provided in Table 4, while the feature importance analysis is illustrated in Figure 5.

**Table 4:** Classification results for the same word

Training Set				Test Set		
Category	Precision	Recall	Recall	Precision	Recall	Recall
Oki	1.00	1.00	1.00	1.00	1.00	1.00
Samsung	1.00	1.00	1.00	1.00	1.00	1.00
Canon	1.00	1.00	1.00	0.78	0.88	0.82
Epson	1.00	1.00	1.00	0.86	0.75	0.80
Xerox	1.00	1.00	1.00	1.00	1.00	1.00
Average	1.00	1.00	1.00	0.93	0.93	0.92

### Ablation study and model performance validation

**Figure 6:**
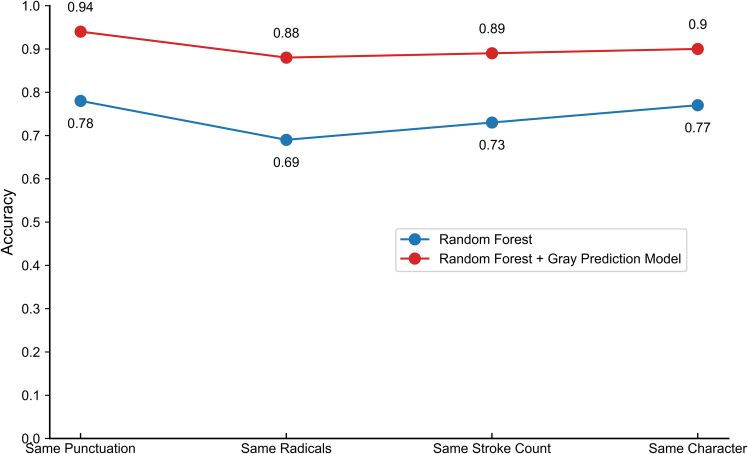
Comparison of algorithmic accuracy: RF vs. GM(1,1)-RF (with trend augmentation) on various tasks

To validate the effectiveness of the proposed GM(1,1)-RF hybrid model, we conducted an ablation study by comparing its performance against the standard RF model without the gray predictive feature enhancement. The results of this comparison are presented in detail in Figure 6.

As shown in Figure 6, the GM(1,1)-RF hybrid model consistently achieves higher accuracy and stability across all classification tasks compared to the standard RF algorithm alone. The experimental results demonstrate that the integration of the gray prediction model provides a significant advantage, substantially improving the overall classification accuracy. This is particularly evident in categories with high inter-class similarity, where the enhanced features provide more discriminative power. The superior performance of the hybrid model validates the effectiveness of the gray prediction feature enhancement strategy, confirming that it is a crucial component for improving model performance, especially in the context of small-sample forensic analysis.

## Discussion

In this study, we proposed a method for the classification of laser printer brands by combining a RF classifier with a GM(1,1) gray prediction model. Our findings demonstrate that the proposed feature set and classification model can effectively distinguish between different printer brands by analyzing microscopic features extracted from printed characters, achieving a high degree of classification accuracy. The feature importance analysis confirmed that parameters such as gray average, area, and RMS line width are highly discriminative for this task. Furthermore, the integration of our Mean reconstruction method proved effective in creating a robust brand signature that mitigates variations from individual device wear.

A key strength of our approach is its strong performance across a variety of classification tasks. For punctuation marks, which have relatively simple structures, the model achieved near-perfect classification for most printer brands, with precision and recall scores of 1.00. Similarly, for Chinese characters with a low stroke count, the model maintained high discriminative power. This success underscores the efficacy of the 33 ISO/IEC 24790-compliant features in capturing stable, printer-specific variations in toner deposition, edge formation, and particle morphology. The feature importance analysis consistently highlighted parameters such as gray average, area, RMS line width, and roundness, confirming that toner density, spatial coverage, line consistency, and particle shape are primary indicators of a printer’s unique physical characteristics. This aligns with the fundamental principles of printing physics, where variations in the photoreceptor drum, laser modulation, and fuser assembly inevitably produce such microscopic deviations.

Despite the model’s overall high performance, we observed a minor decrease in classification accuracy for certain specific scenarios. For instance, the F1-scores for Canon and HP printers were slightly lower when classifying both punctuation marks (0.82 and 0.80, respectively) and multi-stroke Chinese characters (as low as 0.55 and 0.44). This performance degradation can be attributed to several factors. Firstly, the high similarity in printing mechanisms and toner formulations among certain consumer-grade printer brands (like Canon and HP) can result in highly overlapping feature distributions, especially for complex characters. As the number of strokes in Chinese characters increases, the character’s structure becomes more intricate, which appears to dilute the discriminability of individual features and introduces challenges for the model. Secondly, an uneven distribution of data samples in the training set for some categories may have limited the model’s ability to generalize effectively, leading to lower recall for those specific brands. These observations highlight a critical challenge in forensic document analysis: the trade-off between feature complexity and inter-class similarity.

This research provides a valuable and practical solution for the forensic examination of printed documents, laying a solid foundation for future advancements in the field. Building upon the limitations identified in this study, future work should focus on further enhancing the model’s performance and robustness. Key directions include expanding the feature set to incorporate more complex characteristics like stroke curvature and texture complexity, optimizing the feature selection process, and enlarging the experimental sample to include a more diverse range of printer models and environmental conditions.

### Limitations of the study

We acknowledge several limitations in the current study that offer clear avenues for future research.

First, while our discussion on the temporal stability of features is supported by strong evidence from existing literature, this study did not include a long-term longitudinal analysis tracking the feature evolution of the specific printer models used.^16^ Such an investigation would provide invaluable direct data on the rate of feature drift and further validate the method’s forensic applicability over extended periods of use and aging.

Second, the dataset, while sufficient to demonstrate the method’s viability with 14 printers from five major brands, is not exhaustive. The generalizability of the model could be further enhanced by expanding the experimental sample to include a broader range of brands, a greater number of models within each brand, and printers from different production batches. Furthermore, the impact of varying environmental conditions, such as temperature and humidity, on classification performance was not explicitly investigated.

Finally, our feature set was curated based on established forensic principles and parameters defined by the ISO/IEC 24790 standard. While effective, this approach may not capture all possible discriminative information. Future work could focus on optimizing the feature subset, for instance by applying recursive feature elimination and exploring additional complex features, such as stroke curvature and texture complexity, to further improve classification accuracy.

## Resource availability

### Lead contact

Further information and requests for resources should be directed to and will be fulfilled by the lead contact, Yinxuan Qu (20196852@ppsuc.edu.cn).

### Materials availability

This study did not generate new unique reagents.

### Data and code availability

• The raw numerical data reported in this paper are openly available in Zenodo and can be accessed at https://doi.org/10.5281/zenodo.17258957. The accession number (https://doi.org/10.5281/zenodo.17258957) is listed in the key resources table.
• The original code for data analysis is openly available in Zenodo and can be accessed at https://doi.org/10.5281/zenodo.17258957. The accession number (https://doi.org/10.5281/zenodo.17258957) is listed in the key resources table.

## Acknowledgments

The manuscript was supported by the Fundamental Research Funds for the Central Universities of People’s Public Security University of China (no. 2020JKF502) and by the Key Laboratory of the Questioned Document Examination (China Criminal Police University), 10.13039/501100010868Ministry of Public Security of China (2022KFKT03).

## Author contributions

Y.Q. designed and manufactured the experimental samples. Y.Q., C.L., and C.Y. analyzed and processed data. Y.Y. was responsible for methodology. C.Y. and C.L. were responsible for writing original draft. Y.Q. and Y.Y. were responsible for writing – review and editing.

## Declaration of interests

The authors declare no competing interests.

## STAR★Methods

### Key resources table

**Table:** 

REAGENT or RESOURCE	SOURCE	IDENTIFIER
**Deposited data**		

Raw numerical data and source code for analysis	This paper	https://doi.org/10.5281/zenodo.17258957

**Software and algorithms**		

Python (Version 3.9 or later)	Python Software Foundation	https://www.python.org/
scikit-learn	Pedregosa et al.^17^	https://scikit-learn.org/
Numpy	Harris et al.^18^	https://numpy.org/
Pandas	The pandas development team^19^	https://pandas.pydata.org/
ImageXpert image quality measurement systems	ImageXpert Inc.	N/A
python-docx	Steve Canny	https://pypi.org/project/python-docx/

**Other**		

EPSON EXPRESSION 10000XL	Epson	N/A

### Method details

#### Experimental sample preparation

In this experiment, 2,500 commonly used Chinese characters, 1,000 less frequently used Chinese characters, as well as common punctuation marks and letters were selected as the experimental samples. The font used was Song Ti No. 4, with black text color and no background color.

To ensure the representativeness of the samples, 14 sets of laser printers from five different brands were used, with each printer continuously printing until the printed text became unrecognizable. The printing paper used was 70g/m^2^ A4 paper from a uniform brand to ensure repeatability and consistency of the samples.

**Figure 7:**
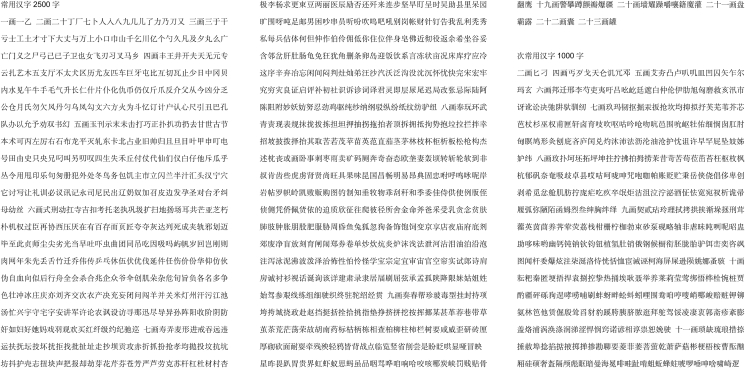
Example of experimental sample design

In terms of character measurement, experimental samples were selected from pages 200 to 500. To ensure controllability and stability, the selection process considered factors such as high usage frequency, stable morphology, and strong structural representativeness. Characters were chosen in three distinct categories—identical characters, identical stroke counts, and identical radicals—along with straight lines and punctuation marks for measurement and analysis. This design allows for a comprehensive evaluation of the model’s performance in handling diverse characters and text types.The experimental samples designed are shown in Figure 7 and Table 5.

**Table 5:** Selection of characters for experimental samples

Same word			
Same number of strokes	Fewer strokes	2 strokes	
		3 strokes	
		4 strokes	
	Multiple strokes	11strokes	
		12strokes	
		13strokes	
Same radicals	“口”		
	“亻”		

Our selection of 14 laser printers from five brands (HP, Canon, Xerox, Samsung, OKI) is grounded in the forensic principles of class and individual characteristics.^20^^,^^21^ Class characteristics—stable features shared by printers of the same brand due to proprietary technologies like toner formulation and fuser design—provide a robust basis for brand-level classification. Conversely, individual characteristics are unique defects arising from manufacturing tolerances and operational wear, such as streaks from a scratched photoconductor drum. By sampling multiple units per brand, our experimental design ensures the model learns the generalizable class characteristics for brand identification, while mitigating the risk of overfitting to device-specific individual characteristics. This approach enhances the model’s robustness for real-world forensic applications. The specific brands and models of the laser printers are detailed in Table 6.

**Table 6:** Basic information on experimental laser printers

Number	Brand	Type	Quantities	Place of origin
1	HP	P1108	4	Vietnam
2	Canon	lpb6018	4	Vietnam
3	Xerox	p158b	2	China
4	Samsung	m12162g	2	China
5	Oki	b4400	2	China

#### Image acquisition and preprocessing

All documents were printed and scanned within a controlled laboratory environment where temperature and humidity were consistently maintained, ensuring uniform experimental conditions across all printer models. The IX system was employed to measure the selected text characters. The process began with scanning the print file as a text image, using a scanning resolution of 800 dpi. During individual character measurements, a consistent ROI (Region of Interest) size was maintained, with the threshold set to 110. To minimize measurement errors, characters from the same position across different print files were consistently selected for measurement. Additionally, multiple measurements of the same character were conducted, and the average value of these measurements was taken as the final data.

**Figure 8:**
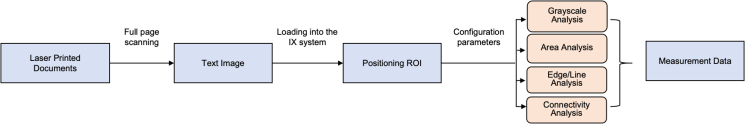
Data acquisition flowchart

The experimental procedure is illustrated in Figure 8.

#### Feature extraction

To construct a robust dataset for printer traceability, this study performed objective quantification of micro-morphological signatures in laser-printed documents. The experimental framework employed the ImageXpert Document Examination System (IX System; ImageXpert Inc.), a methodology compliant with the ISO/IEC 24790 standard.^22^

The selection of features was rigorously grounded in laser printing physics. The interplay of printer components (e.g., laser diode, photoreceptor drum, fuser assembly) generates unique sub-visual micro-variations in toner distribution, edge definition, and particle morphology. These imperceptible deviations serve as device-specific “fingerprints.” Based on this principle, we extracted 33 ISO/IEC 24790-defined parameters across four forensic-relevant categories, as summarized in Table 7.

**Table 7:** Experimentally measured feature parameters

Measurement content	Parameterization	Physical Interpretation
Gray Scale analysis	Gray Average、Gray StDev、Contrast Value、Profile StDev、Profile Gray Min、Profile Gray Max、Profile Gray Avg、Rise Time (Histogram)、StDev of Dark Streaks、StDev of Light Streaks、Gray Av. Dark Streaks、Gray Av. Light Streaks、Focusing、Distribution Symmetry	Toner density distribution consistency
Area analysis	Area、Area Coverage Ratio	Spatial deposition efficiency
Edge/Line analysis	Average Gradient、Line Area、Line Width、 StDev Line Width、RMS Line Width、StDev Deviation、RMS Deviation	Optical system precision
Connectivity analysis	Roundness、Major Axis Length、Minor Axis Length、Perimeter Length、Average Radius、Length (rotated)、Width (rotated)、Axis ratio、Horizontal Extent、Vertical Extent	Toner particle morphology and bonding quality

The ISO/IEC 24790 standard provides the rigorous, device-independent framework for objectively measuring these specific morphological attributes. It defines precise methodologies ensuring the extracted features are quantifiable, reproducible, and directly linked to the physical printing mechanisms.The validity of this standard for revealing printer-specific signatures has been demonstrated in applications like graininess analysis^4^ and edge-feature-based discrimination.^23^

#### Definitions of key feature parameters

To ensure methodological transparency and reproducibility, key non-trivial parameters are defined as follows:

Contrast Value: Measures the contrast degree of pixels within ROI that meet the set polarity and threshold. The line contrast measurement in IX is calculated based on the gray scale histogram, and the calculation formula is shown in Equation 1:

(1) $$\text{Contrast}=\frac{I_{\max}-I_{\min}}{I_{\max}+I_{\min}}\times 100\%$$

##### Rise time (histogram)

A measure of edge sharpness, defined as the number of pixels in the intensity transition zone between the foreground and background. A schematic is provided in Figure S1.

##### Edge and line definitions

Within this framework, an “edge” is defined as a single transition between intensity levels, while a “line” is a composite of two opposing edges. This distinction is illustrated in Figure S2.

##### Connectivity definitions

An 8-connectivity algorithm was used to identify distinct objects. A “Part” was defined as a contiguous region of foreground pixels. A “Hole” was a region of background pixels fully enclosed within a single Part. Examples are illustrated in Figure S3.

However, due to variability among different printing devices, directly using the raw features may reduce the model’s generalization ability. To address this, further data standardization and feature optimization were applied.

#### Mean reconstruction method for brand signature

To reduce randomness among different printers and enhance the stability and distinguishability of the data, this experiment uses the mean reconstruction method to optimize the features of printers from the same brand.

Suppose the original dataset for the jth feature is:

(2) $$X_{j}=\left\{x_{1j},x_{2j},...,x_{nj}\right\}$$

where $x_{\text{ij}}$ denotes the measured value of the ith printer on feature j, and is the number of samples of that printing device. The feature values after mean reconstruction are calculated as follows:

(3) $$\bar{X_{j}}=\frac{1}{n_{j}}\sum_{i=1}^{n_{j}}x_{ij}$$

That is, for all printers of the same brand, the mean value of each feature dimension is computed and used to replace the original data. This approach effectively reduces the randomness introduced by individual devices, enhances overall data stability, and mitigates the impact of outliers on the model.

This method is crucial for addressing intra-brand feature consistency, a significant challenge as raw data from individual printers contains both stable brand signatures (class characteristics) and device-specific noise (individual characteristics). Direct use of this raw data could cause the classifier to overfit to these individual variations. Therefore, the mean reconstruction method acts as a deliberate strategy to isolate the stable brand signature, a concept central to forensic document examination. By averaging feature values across all printers within a brand, we effectively suppress stochastic, device-specific variations and construct a single, more robust feature vector that represents the brand’s fundamental class characteristics. This process ensures our model learns a generalizable “brand-level pattern”, enhancing its utility for forensic cases where the specific source model is unknown.

The processed dataset is then utilized for feature enhancement in conjunction with a grey prediction model (GM(1,1)), further optimizing the model’s performance, particularly in small-sample scenarios.

#### Gray predictive feature enhancement

##### Introduction of GM(1,1) model

In small-sample environments, data sparsity can hinder classifiers from effectively learning deep feature patterns, thereby impacting classification performance. Most traditional machine learning and deep learning methods require large size training set. Nevertheless, in real examples, the data available for such printer traceability tasks is frequently limited and samples are seldom uniformly spread.

To overcome this kind of challenge, this study comes up with the gray prediction model (GM(1,1)) addressing the problem that series modeling can infer implicit data trends from serialized features. When this is used, therefore, the classification performance is improved especially in small sample case.

The GM(1,1) model is an important predictive model for a grey system theoretical modelling under case of missing data, or small sample size. It helps to enhance the smoothness of the data with the Aid of the Accumulated Generating Operarion (AGO) and incorporate the trends with the exponential fitting.^24^^,^^25^

The data representation is enriched by augmenting the original feature set with the predicted values calculated from the GM(1,1) model. This approach is intended to improve the robustness and generalization performance of the subsequent classifier by providing it with data that reflects underlying feature trends.

##### GM(1,1) model generation process

The mean reconstructed data for each brand of printers are subjected to Accumulated Generating Operation (AGO) to create a new data series which improves data smoothness.

Assume the mean - reconstructed dataset to be given by:

(4) $$X^{\left(0\right)}=\{x^{\left(0\right)}\left(1\right),x^{\left(0\right)}\left(2\right),...,x^{\left(0\right)}\left(n\right)\}$$

The cumulative generated sequence is obtained by firstly cumulatively transforming the original feature data $x^{\left(0\right)}\left(k\right)$ :

(5) $$X^{\left(1\right)}\left(k\right)=\sum_{i=1}^{k}x^{\left(0\right)}\left(i\right),k=1,2,...,n$$

The operation that performing alleviates drastic fluctuation of the data leading to more consistency with the modeling assumption of GM(1,1) and hence improve the accuracy of the prediction.

Then, in GM(1,1) model, an background value matrix B and target vector Y should be formed.

The background value matrix is defined as follows:

$$B=\left[\begin{array}{c} -0.5\left(X^{\left(1\right)}\left(1\right)+X^{\left(1\right)}\left(2\right)\right)\\ -0.5\left(X^{\left(1\right)}\left(1\right)+X^{\left(1\right)}\left(3\right)\right)\\ ⋰\\ -0.5(X^{\left(1\right)}\left(n-1\right)+X^{\left(1\right)}\left(n\right) \end{array}\begin{array}{c} 1\\ 1\\ ⋰\\ 1 \end{array}\right]$$

$$Y=\left[\begin{array}{c} x^{\left(0\right)}\left(2\right)\\ x^{\left(0\right)}\left(3\right)\\ \vdots \\ x^{\left(0\right)}\left(n\right) \end{array}\right]$$

$x^{\left(1\right)}\left(k\right)$ refers to the sequence of the features after the cumulative generation of the original data and the target vector Y stands for the next ones of the original data.

To derive the prediction equation of the GM(1,1), it is necessary to find the model parameters a and b. It can be done with Least Squares Estimation (LSE) method.

(6) $${\left[a,b\right]}^{T}={\left(B^{T}B\right)}^{-1}B^{T}Y$$

It has a rate of change given by a and noise (or offsets) of b. Given a and b, a gray differential equation can be presented to predict future feature values.

The enhanced features are finally found as calculated using the GM(1,1) prediction equation^24^:

(7) $${\hat{x}}^{\left(1\right)}\left(k+1\right)=\left(x^{\left(0\right)}\left(1\right)-\frac{b}{a}\right)e^{-ak}+\frac{b}{a}$$

(8) $${\hat{x}}^{\left(0\right)}\left(k+1\right)={\hat{x}}^{\left(1\right)}\left(k+1\right)-{\hat{x}}^{\left(1\right)}\left(k\right)$$

Specifically, ${\hat{x}}^{\left(1\right)}\left(k+1\right)$ and${\hat{x}}^{\left(0\right)}\left(k+1\right)$ denote the predicted value after cumulative generation and the predicted value of the original data, that is the new enhanced features. The enhanced features will be generated and subsequently added to the original feature set so that the classifier having poorer feature differentiation ability can be improved.

##### GM(1,1)-RF hybrid classification model

**Figure 9:**
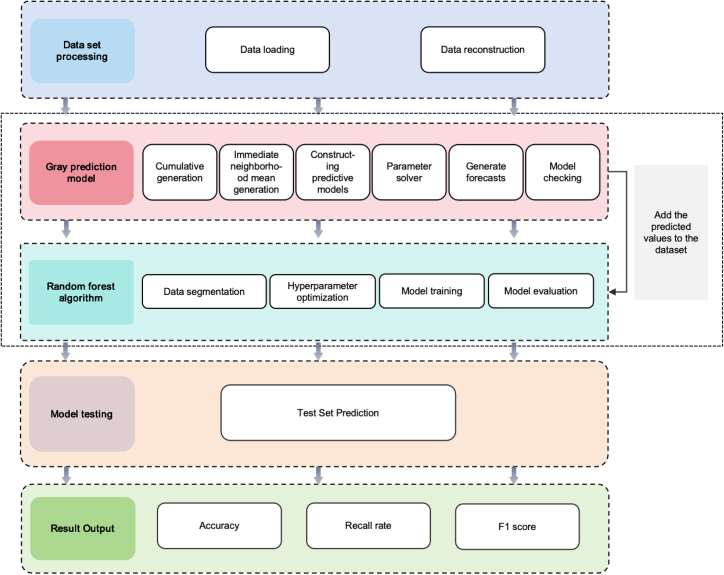
GM(1,1)-RF combined classification model

In this study, a Random Forest classification method (GM(1,1)-RF), which is based on a grey prediction model (GM(1,1)), is proposed for laser printer traceability classification. The method consists of three main stages: data preprocessing, feature augmentation, and classification modeling. The goal of this approach is to improve classification accuracy, enhance model stability, and boost the generalization ability in a small-sample environment. The detailed process is illustrated in Figure 9.

The idea of adding GM(1,1) predictive enhancement features is integrated to the GM(1,1)-RF hybrid model in this paper with the purpose of further improving classification accuracy in small sample regimes.

Random Forest (RF) algorithm is a high-quality ensemble learning method that generates multiple decision trees and aggregates them in order to produce more accurate and stable classification and regression. It is also known to be overfitting and data noise robust as well as it is also strong.^26^^,^^27^^,^^28^^,^^29^

However, Random Forests outperforms a single classifier at processing high dimensional data and provides a quantitated measure of feature importance, thus providing more interpretability of the model.

A systematic training process is designed so that the GM(1,1)-RF model can achieve optimal performance. First, in data preprocessing stage, Mean Reconstruction is carried out to improve data stability and eliminate the difference due to individual same brand printers. Additional features are then generated through GM(1,1) Forecast-Based Feature Augmentation for time-series modeling, optimizing the data representation.

Then, the dataset is split into a 70% training dataset and a 30% test dataset so that the model can generalize to any unseen data effectively.

At stage of model optimization, Grid Search with 5-Fold Cross Validation is used for hyper parameter tuning. The optimized key parameters are number of trees (n_estimators), maximum depth (max_depth) and maximum number of features (max_features) which renders the classifier better. The performance of the trained model on the test set was evaluated using standard classification metrics, including Precision, Recall, and F1-Score for each class.

### Quantification and statistical analysis

The performance of the final classification model was evaluated on the unseen test set using several standard metrics. These included the overall accuracy, and the per-class Precision, Recall, and F1-Score, which were derived from the resulting confusion matrix. All statistical analyses and model implementation were performed using Python (Version 3.9 or later) and standard libraries including scikit-learn,^17^ numpy,^18^ and pandas.^19^ Feature importance scores were calculated from the trained Random Forest model using the mean decrease in impurity (Gini importance) method. This allowed for the identification and ranking of the most discriminative features for the brand classification task.
