# Supplementary material for: Laser-printed document classification using random forest and gray prediction models
Source: iScience. 2025 Nov 21;28(12):114131. doi: 10.1016/j.isci.2025.114131 (PMC12757639; doi:10.1016/j.isci.2025.114131)
Supplement: Document S1. Figures S1–S3 [file mmc1.pdf]

iScience, Volume *28*

Supplemental information

Laser-printed document classification using random forest and gray prediction models

Yinxuan Qu, Chenyang Yu, Chunhui Li, and Yuzhu Yang

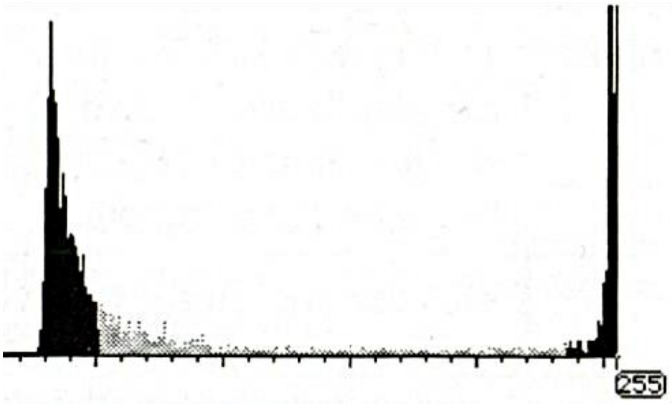

Figure S1. Schematic diagram of rise time histogram

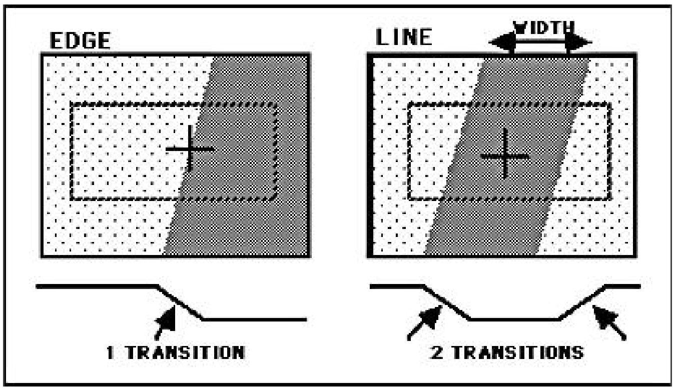

Figure S2. Schematic diagram of edges and lines

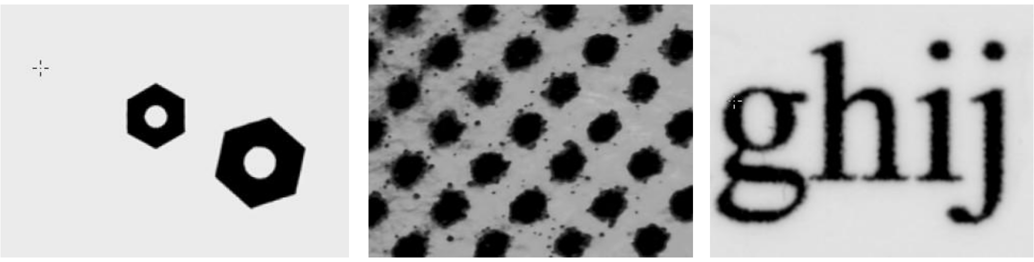

A.) A hexagonal nut as a Part with a central Hole

B.) A solid dot as a Part with no Hole

C.) Characters demonstrating multiple

Figure S3. Schematic diagram of "parts" and "holes"
